# TV Viewing, Independent of Physical Activity and Obesogenic Foods, Increases Overweight and Obesity in Adolescents

**DOI:** 10.3329/jhpn.v31i3.16825

**Published:** 2013-09

**Authors:** Saeid Ghavamzadeh, Hamid Reza Khalkhali, Mohammad Alizadeh

**Affiliations:** ^1^Food and Beverages Safety Research Center, Department of Nutrition, School of Medicine; ^2^Department of Epidemiology and Biostatistics, School of Public Health, Urmia University of Medical Sciences, Urmia, Iran

**Keywords:** Adolescents, Obesity, Overweight, TV viewing, Iran

## Abstract

The aim of this study was to estimate the prevalence of overweight and obesity (OAO) and associated risk factors in a representative sample of students aged 11-20 years in Urmia, Iran. In this population-based cross-sectional study, a multistage random cluster-sampling method was used, through which 2,498 students were selected. OAO were defined based on criteria set by the US Center for Health Statistics in collaboration with the US Center for Chronic Disease Prevention and Health Promotion under the Centers for Disease Control and Prevention (CDC). OAO risk factors were assessed using a questionnaire containing questions about TV viewing, nutrition, physical activities (PA), social and economic factors. Contents of the questionnaire were validated by calculating the content validity ratio (CVR) and content validity index (CVI), based on the responses elicited from 15 experts. Reliability of the questionnaire was obtained from a test and re-test of the questionnaire completed by 15 students. To analyze the data, χ^2^-test, *t*-test, and multiple logistic regression analysis were conducted. The prevalence of OAO was found to be 14.1% among the 11-20 years old students of junior and senior high schools. The results of multiple logistic regression analysis indicated that the educational level of mothers, type of school, and the time spent on viewing TV were associated with an increased risk of OAO while obesogenic foods and PA had no effect on the frequency of OAO [Odds ratio (OR) for the time spent on watching TV one hour more than usual equals 1.27 at p=0.001]. The direct correlation between TV viewing and OAO, which is independent of PA and obesogenic foods, needs to be carefully investigated through randomized clinical trials and cohort studies.

## INTRODUCTION

With the increase in the prevalence of obesity among the adolescents worldwide, the same in Iran has been doubled during the past two decades (1,2). Childhood obesity is linked to diabetes, asthma, and sleep disorders, and obesity in adults is linked to a higher rate of mortality. Longitudinal studies have shown that overweight children are more likely to become overweight adults (3). Overweight in adults will increase the risk of cardiovascular disease, hypertension, gallbladder disease, diabetes mellitus, atherosclerosis, gout, arthritis, and some malignancies (4).

Most of the studies conducted on different adolescent population groups have found a relationship between adiposity and some risk factors associated with nutritional, physical and socioeconomic factors. Some of the identified risk factors are: increase in the consumption of fat (5,6), high consumption of energy-rich foods and alcohol (7,8), low intake of milk and milk products (9), unhealthy dietary patterns (5,7,10,11), skipping the breakfast (5,8,12,13), having been formula-fed instead of being breastfed in infancy (14), watching TV (15-19), physical inactivity (7,8,11,20), insufficient sleep duration (7), high BMI in parents (11,20-23), and high birthweight (11,21). However, the mechanisms through which TV viewing affects overweight remain controversial. It has been proposed that viewing TV increases overweight because, while watching TV, people spend less time on performing PA and, at the same time, they increase their consumption of obesogenic foods (18). Nevertheless, Ekelund found that viewing TV increases adiposity independent of physical activity (24). In the present study, we tried to investigate TV viewing, the level of PA, and the consumption of obesogenic foods to find any possible relationship with overweight and obesity.

To find out a practical solution to OAO in adolescents in Urmia, first we have to know the severity of the problem and the risk factors affecting it. The increasing prevalence of OAO among adolescents has necessitated studying a range of related risk factors simultaneously. Overweight is the result of genetic and environmental factors (25). The tripled increase of overweight in the last 3 decades (26) implicates that environmental factors have been more influential compared to genetic factors.

Identifying the major cause of obesity from among all the environmental factors in any population will be interesting. Therefore, we aimed to investigate the prevalence of OAO and its nutritional, physical, social and economic risk factors in a representative sample of male and female students aged 11-20 years in Urmia.

## MATERIALS AND METHODS

The study was conducted in accordance with the guidelines laid down in the Declaration of Helsinki, and all procedures involving human subjects were approved by the Ethics Committee of Urmia University of Medical Sciences. Moreover, written informed consents were obtained from all the participants.

This population-based cross-sectional study was performed between December 2008 and March 2009 on junior and senior high school students aged 11-20 years in Urmia at the centre of West Azerbaijan province in northwest of Iran. The participants were selected through a multistage random cluster-sampling technique. In doing so, all schools of Urmia city (girls and boys schools, public and private schools) were listed. Then, 29 schools from among all the 375 junior and senior high schools were selected using a table of random numbers. It should be noted that, after obtaining permission from the Ministry of Education, the aim of the study and its importance were presented to the principals and assistant principals of the selected schools. Next, one class was randomly selected from each grade in the selected schools. In some cases, however, the researchers had to replace that randomly-selected class with another one from the same grade due to the disagreement of a principal to use a specific class because they were behind schedule. At the end, a total of 2,498 students from 92 classes were selected, and all of them were studied.

The researchers instructed all students in filling up the questionnaire. In case of the younger students, however, the researchers themselves filled up the questionnaire with information elicited earlier from those students. In answering some questions, the researchers used information obtained from the parents. In addition to some demographic questions, such as those about name, sex, and the type of school (junior or senior high school), the questionnaire contained the following four groups of questions:

1. Nutritional questions included type of feeding in infancy (breastmilk; formula; boiled cow-, sheep- or goat-milk; breastmilk+others), frequency of breakfast consumption in a week, history of dieting, use of drug or food supplement (oral and non-oral). Information was also collected using qualitative Food Frequency Questionnaire (FFQ) with 6 items regarding obesogenic foods: (i) chips or fried potatoes, (ii) sausages, bologna, and hamburgers, (ii) pizzas, (iv) *Tahdig* (crisp rice, potato, or bread which is taken from the bottom of a pot of rice; it is a specialized Iranian cuisine and is usually greasy), (v) butter, and (vi) nuts.
2. PA-related questions included time spent each week on PA; walking; resting; studying (textbooks and others); attending classes (school, remedial, extracurricular, foreign language, computer, etc.); viewing TV, video; working with computers, play stations; and playing sitting games (chess and dominoes).
3. Social questions included family-size, birth order of the children, parents’ educational level (non-literate; elementary, junior high school, senior high school, or university education), parents’ job (unemployed, retired, government employee, non-governmental employee/employer, and having both governmental and non-governmental jobs).
4. A question was also intended to identify the economic status of the students’ families. The level was determined based on the type of school (i.e. public or private) that the student was studying in.

After eliciting responses from 15 experts on the contents of the questionnaire, it was validated by calculating the content validity ratio (CVR) and content validity index (CVI). The content validity ratio was calculated applying the following formula:

CVR=(NE-N/2)/(N/2), where

N=All the experts, and NE=Number of experts who chose “the question is essential”; the other two options were: “the question is useful but non-essential” and “the question is non-essential.”

Next, the calculated CVR was compared with the Lawshe's table. Questions which had a higher value than that in the table were considered valid, and the other questions were omitted.

Moreover, the content validity index for each question was calculated using the following formula:

CVI=Number of experts who gave a score of 4 or 3 from among the scores 4, 3, 2, and 1 to a given question/Total number of experts.

The questions with CVI values greater than 0.79 were considered valid.

The reliability of the questionnaire was obtained through a test and re-test process, in which 15 students took part. A high degree of correlation was found among the responses to the tests (r=0.83).

The following are the two processes involving measurement of the participants’ weight and height:

*Weight measurement*: Weights of the participants were measured (with an accuracy of 100 g) with least clothing (i.e. without shoes, coats, overcoats, and any heavy things, such as the phone and keys in their pockets). A digital scale (Tefal, France) was used, with their body having no movement. For more precision, the students were instructed not to look down at the scale. This was because looking down at the scale could result in an error of approximately 100 g. The students were also asked not to hold anything while they were being weighed. In those cases where any one of the above principles was not followed, the student was weighed for a second time. In each case, the scale was set at zero before weighing again.

*Height measurement*: Height of the participants was measured using a measuring tape. The students were asked to take off their shoes. The accuracy of measurement was up to about 0.5 cm. The students were asked to press their heels and legs together against the wall near the tape and to keep still during the measurement.

Height measurement was performed with one of the researchers standing at the left side of the participants taking their chins with his/her left hand and holding their heads on Frankfurt horizontal plane, a standard craniometric reference plan passing through the right and left porion and the left orbitale. To eliminate the effect of hair thickness on the measurement, the researcher measured their height while holding a large triangle by the right hand and pressing it lightly on the sagittal plane of the participants’ heads. When any one of the principles of measurements was not applied successfully, the height measurement was repeated.

Body mass index (BMI) was calculated using the following formula:

BMI=Weight in kg/(Height in metre) ^2^

Overweight, obesity, and underweight status were determined in accordance with criteria developed through the US Center for Health Statistics in collaboration with the US Center for Chronic Disease Prevention and Health Promotion under CDC: participants with BMI >85^th^ percentile but ≤95^th^ percentile were considered overweight, those with BMI >95^th^ percentile were considered obese, and those with BMI <10^th^ percentile were considered underweight.

### Statistical analysis

In some sets of filled-up questionnaire, the sum of time spent on PA and time of inactivity was more than 24 hours. Therefore, the researchers eliminated them from the analysis; χ^2^-test and *t*-test were applied to assess the differences between OAO and the normal-weight groups. Furthermore, to estimate the effect of each variable on OAO, a multiple logistic regression analysis was conducted after modifying the effect of the other variables. The analyses were performed using SPSS (version 13.0) (SPSS, Chicago, IL, USA), and p<0.05 (two-tailed) was considered significant.

## RESULTS

In this research project, a representative sample consisting of 2,498 students aged 11-20 years (14.52±1.71) was selected. The prevalence of OAO and underweight among the students was found to be 14.1% (n=352) and 7.6% (n=190) respectively, and the remaining students (i.e. 78.3%, n=1,956) were in the normal range of BMI. Since the aim of this research was to compare the two groups: overweight and obese, at this point, the underweight students (n=190) were excluded from the analysis. Consequently, the statistical analysis was performed on 2,308 students. From among these students, 1,323 (57.3%) had been selected from the junior high schools and the remaining 985 students (42.7%) from the senior high schools. It was found that the prevalence of OAO among junior and senior high school students (after excluding the underweight students) was 13.9% and 17.1% respectively. The prevalence of OAO among boys and girls (after excluding the underweight students) was 16.4% and 14.2% respectively.

The variables whose relationship with the frequency of OAO among the participants was examined are presented here.

*Nutritional variables*: This study found no significant differences between the OAO group and the normal-weight group in terms of the type of feeding in infancy (breastmilk; formula; boiled cow-, sheep- or goat-milk; breastmilk+others), frequency of breakfast consumption in a week, history of dieting, drug or food supplement intake (oral and non-oral), and the items included in the Food Frequency Questionnaire (FFQ).

*Physical activity variables*: The results obtained from independent *t*-test indicated that the mean of time spent on walking (p=0.037), viewing TV (p=0.0001), and physical inactivity (p=0.0001) were significantly different among the OAO students and the normal-weight students (Table 1).

*Social variables*: The results of data analysis revealed that the parents’ educational level and mothers’ job (housewife or employed) played a significant role in the frequency of OAO among the students. No association was, however, found between the other social variables and frequency distribution of OAO among the students. The effect of the educational level of parents on distribution of OAO has been shown in Table 2. The frequency of OAO was higher among the students whose parents had higher education. Furthermore, as it can be seen in Table 3, the frequency of OAO among children whose mothers were employed was found to be higher as well (p=0.047).

*Economic variables*: The frequencies of OAO among students of public schools and private schools were significantly different. As Table 4 suggests, students in the private schools had a higher frequency of OAO than students in the public schools (p=0.001).

To assess the concurrent effect of each variable on the frequency of OAO, the OR for each variable was calculated through applying a multiple logistic regression analysis. After controlling effects of the other variables, the relationship of the mothers’ job and the educational level of fathers with the frequency of OAO among children disappeared. However, the relationship between the educational level of mothers (reference: non-literate, elementary: OR=1.47 and p=0.046, junior high school: OR=2.05 and p=0.001, senior high school: OR=2.25 and p=0.001, university: OR=2.39 and p=0.007), type of school (reference: public schools, private schools: OR=1.85 and p=0.001) and time spent on TV viewing (OR=1.27 for time spent on watching TV one hour more than usual and p=0.001), and the frequency of OAO remained significant (Table 5).

## DISCUSSION

In the present study, the prevalence of underweight and OAO in Urmia was found to be 7.6% and 14.1% respectively based on criteria set by the CDC. The prevalence of underweight and OAO was 13.9% and 8.82% respectively as found in a study by CASPIAN (the only national survey among Iranian children and adolescents performed in 2003-2004) in accordance with the same criteria (1). The prevalence of underweight and OAO in Urmia is siginificantly different from that at the national level. The prevalence of OAO in Urmia was similar to that in the cities of Tabriz (14%, using criteria set by IOTF) (27), Isfahan and Arak (13.6% among girls and 9.3% among boys, using criteria set by CDC) (28). Also, the prevalence of OAO in Urmia was similar to that among a representative sample of all ethnicities living in Iran (12.5%, using criteria set by IOTF) (29). The prevalence of OAO in Urmia was more than that in Kerman (7.3%, using criteria set by CDC) (30) and Birjand (8.4%, using criteria set by CDC) (31). The prevalence of OAO in Urmia was less than that in Tehran (29%, using criteria set by CDC) (2) and Rasht (27.2% of girls, using criteria set by IOTF) (32). The differences between the cities of Iran, regarding the prevalence of OAO, could be related to the socioeconomic status (SES) of people and their culture, behaviour, race, PA, dietary patterns, and lifestyle.

**Table 1.** Comparison of time spent per hour on physical variables in OAO group and normal-weight students group by independent *t*-test in Urmia

Activity	Normal weight		Overweight and obese		p
	Mean	SE	Mean	SE	
Physical exercise in a week	0.745	0.02	0.712	0.05	0.593
Walking in a day	0.98	0.02	0.87	0.04	0.037
Study in a day	3.29	0.03	3.36	0.08	0.481
Sleep in a day	8.68	0.03	8.72	0.08	0.537
Sitting games in a day	0.24	0.01	0.27	0.02	0.292
Working with computer in a day	0.42	0.02	0.47	0.05	0.26
Watching TV in a day	2.135	0.03	2.501	0.07	0.0001
Class attendance in a day	5.95	0.01	5.99	0.03	0.272
Total time of physical inactivity in a day	20.696	0.06	21.304	0.13	0.0001
Total time of physical activity in a day	1.728	0.03	1.581	0.07	0.089

SE=Standard error

**Table 2.** Effect of parents’ educational level on distribution of OAO in the junior and senior high school students in Urmia

Level of education		Normal weight n (%)	Overweight and obese n (%)	Total n (%)
Non-literate	Father	241 (91.3)	23 (8.7)	264 (100)
	Mother	479 (91.1)	47 (8.9)	526 (100)
Elementary	Father	514 (88.5)	67 (11.5)	581 (100)
	Mother	593 (86.4)	93 (13.6)	686 (100)
Junior high school	Father	384 (82.1)	84 (17.9)	468 (100)
	Mother	354 (81.2)	82 (18.8)	436 (100)
Senior high school	Father	494 (83.2)	100 (16.8)	594 (100)
	Mother	372 (78.9)	100 (21.1)	473 (100)
University education	Father	237 (77.5)	69 (22.5)	306 (100)
	Mother	78 (78)	22 (22)	100 (100)
Total	Father	1,870 (84.5)	343 (15.5)	2,213(100)
	Mother	1,877 (84.5)	344 15.5)	2,221(100)

The frequency of OAO in different educational levels of parents was significantly different in χ^2^-test (p=0.0001)

**Table 3.** Distribution of OAO regarding mothers’ work in Urmia

Working status	Normal weight n (%)	Overweight and obese n (%)	Total n (%)
Housewife	1,778 (85.1)	311 (14.9)	2,089 (100)
Employed	158 (89.8)	40 (20.2)	198 (100)
Total	1,936 (84.7)	351 (15.3)	2,287 (100)

There was significant difference in the distribution of OAO in χ^2^-test (p=0.047)

**Table 4.** Distribution of OAO in public and private schools in Urmia

Type of school	Normal weight n (%)	Overweight and obese n (%)	Total n(%)
Public schools	1,778 (85.6)	300 (14.4)	2,078 (100)
Private schools	173 (77.2)	51 (22.8)	224 (100)
Total	1,951 (84.8)	351 (15.2)	23.2 (100)

There was significant difference in the distribution of OAO in χ^2^-test (p=0.001)

*Nutritional factors*: This study found no relationship between OAO and the nutritional factors. This might be because the researchers limited the number of questionnaire items to achieve a higher degree of validity and reliability. That is, the researchers sufficed to food items which are more likely to be consumed during TV viewing. Consequently, the FFQ contained only 6 items concerning obesogenic foods, and this may have been the cause of not arriving at insignificant results concerning the relationship between OAO and nutritional factors. Other studies conducted on different populations, which had examined more nutritional factors, showed that OAO has an inverse relationship with some nutritional factors, such as consumption of fruits and vegetables (33), whole grains (34), skim milk and skim milk products (9), healthy dietary patterns (35), low-fat foods (5,6), low-sugar foods (36), having been breastfed in infancy (14), and consumption of breakfast (5,8,12,13) and a direct relationship with consumption of unhealthy dietary patterns (35), high-fat milk and milk products (35), refined grains (34), fatty meats (35), fat (5,6), refined carbohydrates (36), drinks containing sugar (36), and having been formula-fed in infancy (14). This study had limitations in assessment of nutritional factors but found that the factors that were examined had no decisive role in OAO; so, conducting specialized studies in this field is necessary.

**Table 5.** Concurrent association between each variable and OAO estimated by multiple logistic regression model

Variable	Odds ratio	p
Mothers' job		
Housewife	1.00	-
Employed	1.1	0.647
Educational level of mothers		
Non-literate	1.00	-
Elementary	1.47	0.046
Junior high school	2.05	0.001
Senior high school	2.25	0.001
University education	2.39	0.007
Kind of school		
Public	1.00	-
Private	1.85	0.001
Education levels of students		
Junior high school	1.00	-
Senior high school	1.25	0.089
Physical variables		
Studying	1.02	0.069
Sleeping	1.04	0.39
Walking	0.913	0.218
Sitting games	1.24	0.098
Computer-use	1.001	0.991
TV viewing	1.27	0.001
Classroom	1.02	0.819
Total physical activity	0.944	0.371
Constant amount	0.035	0.001

*Physical activity factors*: Results of multiple logistic regression analysis revealed that, contrary to low PA and obesogenic foods, watching TV was a strong risk factor for OAO. Two important mechanisms concerning the effect of TV viewing on obesity have been proposed. The first proposal states that time spent on watching TV reduces the time spent on performing PA. The results of this study which are consistent with the results of study conducted by Ekelund *et al.* (24) showed that viewing TV, independent of PA, increased the risk of OAO. The second proposal states that viewing TV provides time to consume more obesogenic foods. The obesogenic foods under study in the present investigation were those that are easily available and have high calories, such as chips, fried potatoes, sausages, bologna, hamburgers, pizzas, *Tahdig*, butter, and nuts. This study showed that the amount of obesogenic foods consumption was not related to the length of time the adolescents spent on watching TV. It means viewing TV affects OAO, independent of obesogenic foods. Probably, viewing TV acts through mechanisms other than reducing the expenditure of energy (19,24,37) and increasing the consumption of obesogenic foods. These mechanisms probably include the effects of food advertisements on dietary patterns (38), reduction of metabolic rate to lower than that in rest time (39), circulatory cortisol elevation due to excitement (40), and disruption in the circadian rhythm (41). The findings of the present study suggest that watching TV, not PA or obesogenic foods, was associated with OAO in adolescents. Students in Urmia spent the same amount of time on PA and consumed the same amount of obesogenic foods. However, watching TV attributed most to the variance of OAO. To control overweight among children, parents should reduce the time their children spend on watching TV as much as possible.

*Social and economic factors*: Distribution of OAO was significantly different among various levels of social and economic factors. This study showed that the frequency of OAO was higher among those whose mothers had a higher level of education and those who went to private schools. There are some similarities between social and economic factors. Perhaps, it would have been better if we had included more economic factors to find their net effect on the frequency of OAO. If the given social factors in this study were the same as markers of economic status, it would have meant that the frequency of OAO has had a direct relationship with economic status. However, if no correlation was found between social factors and economic status, it would indicate that there was not a direct relationship between social status and the level of health-related knowledge. In such cases, the role of community health authorities becomes more prominent. It seems that various social and economic factors have different effects in different populations. Review of literature showed that age (42), sex (43), educational level of parents (44), race (43), SES (44), the location of the school in the city (45), and the weight of mother (46) were effective in the distribution of OAO. In most studies carried out in developing countries (11,13,22,44,47), it was found that high educational level of parents was a risk factor of OAO for their children. In developed countries, however, it was found to be a protective factor (20,43,48). This issue is almost true for some other social and economic factors, such as mothers’ occupation and income. In developing countries mothers’ occupation and increase in income enhanced the risk of OAO (20,22,48-50). This inconsistency of results among various studies may be due to a variety of reasons: (i) probably, the attitudes of people in developing countries about overweight differs from that of people in developed countries; (ii) the knowledge of appropriate serving-sizes may be limited among people of high SES in developing countries; and (iii) perhaps, in developing countries, overfeeding children is assumed to be a way of parents giving attention to their children.

Obviously, families with moderate and high income only can pay high costs of private schools. If we consider the education of adolescents in private schools as an indication of the high income of their families, the results of this study are consistent with Tremblay's (51). In both the studies, the frequency of OAO and level of income showed a direct relationship.

The strengths of this study lies in its population-based approach and large sample, which resulted in a great reduction of random errors. Another advantage was the precise measurement of weight and height. In addition, this study was highly valid because of its large sample-size, valid and reliable questionnaire, relatively brief questionnaire, and extraction of the net effect of each factor under study.

### Limitations

There were some limitations associated with this study. First, briefness of the questionnaire did not allow the researchers to find all the existing relationships between nutritional factors and the participants’ OAO. Second, due to the school principal's disagreement in a few cases, the researchers had to replace a randomly-selected class with another class which was having a sports exercise. Third, in some sets of questionnaire, the sum of time spent on PA and that of inactivity was more than 24 hours; hence, the researchers eliminated them from the analysis. Fourth, due to some religious considerations, anthropometric measurements were performed by two data-gathering teams: a male team for boys schools and a female team for girls schools. If all anthropometric measurements were performed by one team, the reliability of data would have increased.

### Conclusions

Among the students living in Urmia, educational level of mothers, type of school (public or private), and watching TV have a net association with OAO distribution. The higher educational level of mothers was associated with a higher frequency of OAO. The distribution of OAO was higher in students of private schools than that of the public ones. Time spent on watching TV one hour more than usual increased the risk of OAO 1.27 times. To confirm the effect of TV viewing, independent of PA and obesogenic foods, as it was found in the present study, conducting randomized clinical trials and cohort studies is essential.

## ACKNOWLEDGEMENTS

This work was supported by the Urmia University of Medical Sciences. We thank the participants of this study for their enthusiastic support.
